# The Feminine Condition and Women's Sexual and Reproductive Health in Brazil and France

**DOI:** 10.3389/fpsyg.2022.862431

**Published:** 2022-05-02

**Authors:** Simone Santana da Silva, Cinira Magali Fortuna, Gilles Monceau, Marguerite Soulière, Anne Pilotti

**Affiliations:** ^1^Department of Ed2ucation, State of Bahia University, Senhor do Bonfim, Brazil; ^2^Ribeirão Preto College of Nursing, Universityof São Paulo, Ribeirão Preto, Brazil; ^3^Laboratoire École, Mutations, Apprentissages (EMA), CY Cergy Paris Université, Cergy, France; ^4^Faculty of Social Sciences, University of Ottawa, Ottawa, ON, Canada

**Keywords:** sexual and reproductive health, women's rights, parturition, public health, gender

## Abstract

**Introduction:**

Elements mark the reality of reading the female body in symbolic constructions and social symbols in the exercise of their reproductive health. The study aims to identify elements that characterize the female condition while analyzing the reproductive health of Brazilian and French women.

**Materials and Methods:**

A qualitative, multicenter, international study was conducted in Brazil and in France between 2016 and 2019. Data were produced through the use of semi-structured scripts. Focus group discussions and individual interviews were conducted with women who gave birth, hetero-female couples who lived the experience of gestation and birth of a baby, and professionals of maternal and childcare services or members of the associations concerned with the health of mothers and babies. It was guided by the theoretical-methodological framework of institutional analysis in line with the French Institutional Socioclinics.

**Results:**

Sexual and reproductive health in the realities researched in Brazil and France are sometimes close and sometimes far apart. In what involves the Brazilian health system, abortion is criminalized and often performed illegally. Furthermore, pregnancy, childbirth, and the postpartum period are highly medicalized. In addition, childbirth is not assured as an experience for a woman and her family. This aspect is confirmed by high numbers of cesarean sections performed or by maternal and infant mortality indicators. The French health system prioritizes vaginal deliveries and seems to assure more autonomy to women, but at the same time, it is worn out by the logic of profit, the efficiency of actions, and the rationalization of practices. In association with these, there are other intrinsic elements in the functioning of the institution that delineates the format of each country: notion of women's rights, violence against women, and discussion regarding the oppression markers of race, gender, sexuality, and social class.

**Conclusions:**

Both countries reveal aspects related to the social role of women's bodies. The established logic reflects in the decision to have children, motherhood, women's autonomy over their own bodies, and in the core values linked to the termination of pregnancy and the professional practices developed in prenatal care, childbirth, and postpartum.

## Introduction

The “feminine condition” brought in the present article is primarily underpinned by the approach of philosopher Simone de Beauvoir (de Beauvoir, 1949; Beauvoir, 2016) who betted on the critique of patriarchy^1^and self-criticism of the social spaces occupied by women. We recognize that, through the years, the term is updated in the perspective of a state and is not restricted to a relationship. It broadens the conception of knowledge of oneself and the appropriation of the body by women, motherhood, rights, and reproductive health, which will unfold within the feminist movement and, in some cases, fight for the overcoming of the binary model.

In view of the above, it is understood that the sexual and reproductive health of women, which involves the right of people to decide about sexual practices, self-care, and reproductive planning, is formed from a social, historical, cultural, and economic phenomenon marked by social, gender, ethnic, and racial inequalities. This context does not affect women of different countries and cultures equally.

In global terms, the debate related to women's sexual and reproductive rights is inserted in different international documents, such as, the Convention on the Elimination of All Forms of Discrimination Against Women (CEDAW) (United Nations Human Rights, 1981). There are other elements intrinsic to the reality of each country that demarcate the context of rights, among which are the notion of women's rights, the notion of violence against women, and the discussion of markers of oppression such as racism, sexism, heterosexism, and classicism.

In Brazilian context, women's sexual and reproductive rights are protected by the 1988 Federal Constitution; however, in real terms, public debate seems impossible when it comes to gender issues. Abortion, with a few legal exceptions, is criminalized in Brazil and is largely performed illegally (Diniz et al., 2017; Cardoso et al., 2020). Moreover, pregnancy, childbirth, and the postpartum period are highly medicalized. Childbirth is not assured as an experience for a woman and her family (Betrán et al., 2018; Lansky et al., 2019). Such aspects are confirmed by high numbers of C-sections performed or by maternal and infant mortality indicators. Another very marked element in the Brazilian reality is racism that interferes in people's lives even before they are born, because the color of the skin interferes not only with access to prenatal care but also with the quality of examination performed by a physician. In addition to this reality, there is social inequality that is also reflected in the rates of teenage pregnancy and infant mortality (Rattner and Moura, 2016; Bottallo, 2021).

The French health system prioritizes the performance of vaginal deliveries and proposes to ensure women's autonomy. There is a broad discussion in the country about relationships between women and medical staff, as well as about the evolution of medical practices in the hospital environment over the years. Such practices are usually justified by the idea of safety. At the same time, this system is worn down by the logic of profit, efficiency of actions, and rationalization of practices (Coulm and Blondel, 2013; Césarine, 2017; Silva et al., 2021).

For clarification purposes, the Brazilian health system is decentralized in its management and in existing health policies. Decentralization is one of the organizational principles of the Brazilian Unified Health System. There are, therefore, responsibilities attributed to the Union, states, and municipalities, which need to act in an integrated way. France has a nationally centralized health system, with decentralization only at the management level with regional health agencies.

However, it is inferred the relationship between sexual and reproductive health and the feminine condition. The recognition of birth and the reproductive process as individual and collective sources of women's empowerment, can overcome the interference of the State: through non-discrimination, non-coercion, non-violence, and non-domination of bodies.

The present text proposal uses the theoretical-methodological framework of institutional analysis (IA) to support the argument that articulates the reality of the female condition with sexual and reproductive health in Brazil and France. IA, through its characteristics and devices, questions what seems naturalized in the eyes of the majority (de Rodrigues, 2005; Clarindo et al., 2021). Thus, we started our research from the understanding that reproductive health is an institution constituted of norms and rules legitimated in policies and legislation, being full of instituted protocol practices. The forces called instituting destabilize those instituted in a transformation movement, which is called institutionalization. The articulation of these three movements: instituted, instituting, and institutionalization conforms to the institution (Lourau, 1970). In French Institutional Socioclinics, a continuity approach to IA (Fortuna et al., 2017), the analyzers are the elements that provoke the analysis of institutional contradictions. It is through them that contradictory and usually hidden dimensions are revealed. In view of the above, the feminine condition acts as an analyzer of the reproductive health of Brazilian and French women.

The present production aims to identify elements that characterize the female condition while analyzing the reproductive health of Brazilian and French women. It is an offshoot of a pilot research project funded by the Montfort Research Institute^2^ and has an international multicenter character that had birth and parenthood as central elements in the discussion (Soulière et al., 2020).

## Materials and Methods

### Study Design

The research is based on the interest in developing a collaborative (composed of fathers, mothers, maternal and childcare professionals, and researchers), international, and interdisciplinary (health and social care professionals) pilot project on a subject that could contribute to the debate surrounding birth and parenting.

This is a qualitative study based on interviews with groups (focus groups) composed of health professionals, fathers, and mothers from Brazil, Canada, and France. For the analyses of this article, only group discussions held in Brazil and France were included. In addition to these group discussions, individual interviews were conducted and a research diary was adopted. It was guided by the theoretical and methodological references and the theoretical-methodological framework of institutional analysis (Lourau, 1970) the latter of which is in line with the French Institutional Socioclinics that seek to be attentive to the institutional dynamics and to understand the logic of professional practices implemented (Monceau, 2003, 2018; Silva et al., 2021).

In each country, two groups were organized with professionals from different healthcare facilities for the care of women and babies, as well as two groups with parents from different social contexts. The work with the groups involved the proposition of questions for the creation of a questionnaire with themes about expectations of perinatal care and interventions to be answered in the future by other parents. From the dialogue with the different groups for the formulation of the questionnaire, it was possible to deepen different agendas that gave visibility to those linked, among other things, to the relationship between the feminine condition and women's sexual and reproductive health.

Therefore, focus groups were formed, and each group developed three meetings. The meetings had an average duration of 2 h and were guided by a thematic script. In the intermediary meetings, the aim was to restitute the previous meetings with their respective analyses and to conduct the development of the questionnaire previously mentioned. In the final meetings, the analyses of the intermediary meetings were restituted (Bergier, 2001; Soulière et al., 2020) and the interpretation of the preliminary results was related to the formulation of the questionnaire (Paillé and Mucchielli, 2012). The sessions were recorded and transcribed, and the results were cross-referenced in the preliminary analyses between groups and between countries.

### Period and Place of Research Development

The research was carried out between the years 2016 and 2019 in the city of Ribeirão Preto, SP, Brazil, and in the Hauts-de-Seine and Val d'Oise regions in France.

### Participants, Selection Criteria, and Data Collection Procedures

The study counted the participation of women who have given birth, heterosexual couples who have lived the experience of gestation and birth of a baby, and professionals of maternal and childcare services or members of associations concerned with the health of mothers and babies. It is important to emphasize that the study is based on the perspective of cisgender women (da Silva et al., 2019) as an existence permeated by captures strongly marked by patriarchy and colonization of bodies and thoughts. The emphasis on cisgenerity in the present production is justified by the profile of the women participating in the study, given that the condition of cis-women is often linked to bodies biologically prepared to give birth.

The selection criteria for the participants were interest and availability to participate, people older than 18 years old, and signature on the Informed Consent Form (ICF). Participating mothers and fathers should have experienced pregnancy and childbirth, regardless of the outcome. The professionals, in turn, should have worked in the public services of maternal and childcare.

For the composition of the focus groups, the criteria that will be described below were adopted. Regarding the groups composed of mothers and fathers, two groups were formed by people from different social, economic, and educational backgrounds: “social 1” and “social 2.” In Brazil, they were represented by a public nursery (social 1), which welcomes low-income families in which the majority do not have a college degree, and a language school (social 2), which brings together economically privileged families. In France, the “social 1” group was composed of mothers and fathers using a municipal service, located in a working-class neighborhood, to support families in their parenting, called “*Maison des familles*,” and the “social 2” group was constituted by a parents' association, which was created by middle- and upper-class people to promote the use of a birth house that performs unmedicalized births.

Regarding the group of professionals, two groups of public servers with different profiles were formed, which were classified as “hospital” and “other institution.” The “hospital” group in Brazil was a women's healthcare service whose practices are in line with most hospitals, in other words, with a very high rate of C-sections, and the focus group named “other institution” was constituted by a reference hospital for maternal health that advocates vaginal delivery. In France, the groups were composed of professionals from a maternity hospital and a women's and children's care service called “*Protection Maternelle Infantile*” (PMI). Three meetings were held with each of the groups.

Four focus groups were organized per country composed of groups from 8 to 12 people. Regarding the individual interviews, 9 Brazilian mothers (2 were not part of the focus group), 10 Brazilian professionals (4 were not part of the focus group), 7 French mothers (4 were not part of the focus group), 2 French couples (none participated in the focus group), and 9 French professionals (5 were not part of the focus group) participated.

In relation to the individual interviews, they began with the invitation to the participants of the focus groups. As there was no unanimous agreement to participate in this new moment of the research, participation was requested with an indication of another person with the same profile, that is, belonging to the same social group described above. As the research involves the deepening of different aspects, the individual interviews allowed for a more qualified deepening of aspects that were not addressed in depth in the developed groups.

A semi-structured script was used to manage the groups and conduct the interviews.

### Data Treatment and Analysis

A thematic analysis was performed from Paillé and Mucchielli's perspective (Paillé and Mucchielli, 2012). For the analysis, the analytical process was put into practice as an exercise of qualified, interpretative, and hypothetical analysis.

A hybrid approach was adopted for the analysis, operationalized by a continuous thematization and a sequential thematization. The continuous thematization consists of attributing, regrouping, and adjusting the themes progressively until the end of the apprehensions. The sequential thematization offers support to performing the analysis with distances and approximations between groups.

The steps of the analytical process were transcription of the individual and group^3^ interviews, constitution of the units of meaning and correlation of these with the theoretical references, and reconstitution with the final analyses (Silva et al., 2021).

The research diary notes were cross-referenced with the group summaries and individual interviews. Horizontal syntheses were written for the resulting records from each category of data production, highlighting convergences, divergences, and complementarities.

### Ethical Aspects

To produce the data in Brazil, it was necessary to obtain authorization from the co-participating institutions and approval from the Research Ethics Committee (CAAE n60747716.3.0000.5393).

To ensure the anonymity of the participants, we adopted identifications of the groups and of the individuals interviewed. The identifications of the groups were: “social 1” or “social 2” for the group of parents belonging to the different researched equipment and “hospital” or “other” for the groups formed in the assistance services. We adopted the Arabic numerals “1, 2, or 3” to identify which group meeting was referred to, plus the abbreviation “BRA” or “FRA” to identify the country referred to. The identifications of the individual interviews were: “mother,” “father,” and “PRO” for mothers, fathers, and professionals, respectively, followed by the Arabic numerals “1, 2, or 3” and BRA or FRA.

The individual interviews conducted in France were not transcribed in their entirety. For this reason, the following coding was adopted: a report of individual interview, followed by FRA and mother or PRO and an Arabic number (e.g.,: report of individual interview/FRA/mother/01).

The development of research in France does not require the same ethical procedures as in Brazil. Despite the difference, the research in this country occurred after the authorization of the researched healthcare services and participants. In both countries, bioethical principles were assured, and the signature of the ICF was required. Regarding the parent focus groups formed in France, because they do not meet in health services, and organizational authorization was not requested, only individual consent was requested.

## Results

Although the broad nature of the agenda related to women's sexual and reproductive rights is acknowledged, for the present production, we have valued some elements learned during the research process. In light of the above, once again, we assume that a drawing will be drawn that relates elements of the feminine condition of the countries with aspects linked to sexual and reproductive rights. Thus, Brazil and France revealed approximations and distances related to the social organization in which women belong and in relation to sexual and reproductive health. It is emphasized that this is not a comparative study between the countries but a cross-cutting of distinct realities that allow for the explicitness of aspects less visible and the recognition of specificities and differences. Each reality is made up of particular specificities in the cultural, social, organizational, and political fields, which cannot be compared. In light of this understanding, the study seeks to understand what the Brazilian reality questions the French one and the opposite. This cross-questioning approach has already been successfully used in various doctoral research projects, including, for example, Ribeiro Santana's research on the place of health promotion and disease prevention in the training of nurses in Brazil and France (Santana et al., 2017).

France shows some advances in relation to equal rights between men and women, for example, in representation and economic opportunities, whether in terms of salaries, protagonism, or responsibilities assumed in management positions. In relation to educational aspects, they stand out in the aspects of basic and higher education. In the aspects linked to political emancipation, they stand out in the representativeness in decision-making structures and in what involves elements related to health and survival; they present good indicators of life expectancy.

The data reveal aspects of social demands related to women's roles in society, especially concerning motherhood, as well as female autonomy. We cannot deny the fact that the research itself values the aspects related to parenthood, which tends to emphasize more strongly some elements linked to women's sexual and reproductive rights to the detriment of others. Among these, we can state that we will deal more closely with maternity, abortion, access to information, and violence.

The dialogue centered on the decision to have children appears differently in both countries. In Brazil, the idea of compulsory maternity is much more marked in the discourse of the people interviewed, as can be seen below.

[Interviewee2] even quite newly married, at a week old, people already start asking, “when are you going to have children?”

[Researcher] so there are people who charge...

[Interviewee 2] society, family...

[Interviewee 3] I think that this is cultural, especially for women... she is charged... That she has to be a mother. It doesn't come as a choice, it comes as being a woman... it already determines that I have to have a child (Other - 01/BRA).[Professional 05] I think that we have many cases where the pregnant woman sometimes is not interested. She doesn't want to get pregnant. She gets pregnant to please her partner... this happens a lot

[Professional 01] There is a lot of this! We see it!

[Professional 07] It is to please the partner. Sometimes she already has other children and doesn't want another and sometimes her husband: “no,” the husband wants to try a boy. So, sometimes it is a demand from the partner, isn't it? (Hospital - 01/BRA).[Mother 04] [...] because I never wanted to have a child. In fact, I had them because of him, you know? When I married him, I was in the circus, people... so who lives in a circus never thinks about having a child... I was always traveling, I never had in my head that I was going to have a child, I did not want to, I thought it was unnecessary (Social 1 - 01/BRA).

In France, in turn, the subjective and emotional elements related to the process of becoming pregnant emerge with greater elaboration by the interviewees. These aspects emerge when they talk about the planning processes, preparation for childbirth, and postpartum follow-up existing in the country, the performance of assisted reproduction procedures by professionals, or the legality of interrupting pregnancies, as can be confirmed in the following statements:

[Researcher]: Are these situations in which people are preparing themselves for the process of getting pregnant or actually at the time of childbirth?

[Interviewee A]: In the service (which also offers medically-assisted reproduction/MAR), I am confronted with couples who plan to become parents.... well... the desire to be pregnant, I would say, at the beginning. So, the plan to have a child is very, very early. I'm not saying that other parents, who have spontaneous pregnancies, don't plan and anticipate or... whatever, but for this service, it's something else.

[Interviewee B]: What is particular about this service is perhaps above all that they are... they have already been through the waiting journey that...

[Researcher]: Do you say “waiting journey”?

[Interviewee A]: Yes, because that's often how they present it. They've gone through... Once they had the idea of having a child, they tried naturally, then they went through stimulation and so on, then through.... well... There's everything... exams... well... It's a gradual process... It's a real journey, which they often present as an obstacle course (Hospital - 01/FRA).

When talking about her pregnancy, she said that she was not prepared for pregnancy and that her pregnancy was not planned. She says that before her pregnancy, she was frequently questioned by her gynecologist about her desire to get pregnant. He pointed out that she was over 30 years old. In view of this, she felt pressured by the doctor. [...] She greatly idealized natural childbirth, by vaginal route, with a lot of pain (according to her, it's not a problem) (individual interview report/FRA/mother/02).

There was also the participation of a Brazilian woman living in France who experienced the planning, monitoring of pregnancy, childbirth, and the postpartum period in France. Her opinions clearly demarcate the strength of the culture of practices adopted in her country of origin and give visibility to a tension resulting from the different contexts experienced by this woman in a kind of “clash” of cultures. Although she experienced vaginal birth in France, she clearly demarcates the logic of her culture of origin.

Many people that I talk to who are French, they don't understand why some women prefer the cesarean intervention. So I think that the medical staff does not understand if you request a cesarean section. They even know that, culturally in Brazil, we do a lot of surgical deliveries, but they don't understand why and think it's cool. [...] To be very honest. If I had had a child in Brazil, I would have chosen to have a cesarean section (Mother/FRA/03).

Voluntary interruption of pregnancy is also a prominent element in the analyses of this study. This occurs because it is related, among other things, to the autonomy of women over their bodies, the power of patriarchy and religions in society, besides reflecting on health indicators, after all its illegality is, for example, a major cause of maternal death.

In Brazil, the theme related to the voluntary interruption of pregnancy is quite controversial and often veiled by society. The following statements reinforce this reality:

[Professional 05] It is different in Brazil, which is not legalized and increases the number of abortions.

[Professional 01] High incidence of induced abortion! When we receive a pregnant woman having an abortion here, we don't look too closely because otherwise we have to file a police report and end up at the police station.

[Professional 01] So, we don't discuss much with the person who has an abortion. They come in having an abortion, I take care of it, that's it. I don't look too much into whether she provoked it, if she didn't [...] they go to the shopping center down there, in front of the market, where they have people to sell. So, they go there, get it, use it and come here bleeding.

[Professional 1] is, sometimes they use, come with bleeding to see what happened, if they do the ultrasound everything is fine, they disappear. In a little while, they come back having abortions...because they keep trying until they get them. This is really our reality (Hospital - 01 BRA).

On the contrary, in the French context, the termination of pregnancy is legal, which promotes certain naturalization of the process adopted by women and a more open dialogue about its existence. Furthermore, the access and normalization of abortion facilitate and present the issue of the desire for a child as an important fact for the wellbeing of the mother and the child to come.

[Interviewee C]: It is true that we often ask ourselves the question, when we want to talk about a situation, if it was a desired pregnancy, an accepted or not accepted surprise pregnancy...

[Researcher]: Can you tell me the words? “Desired”...

[Interviewee C]: That's right. Is it a desired pregnancy? Is it a surprise pregnancy?

[Interviewee D: Is this an unplanned pregnancy?

[Interviewee C: An unforeseen pregnancy, exactly.

[Researcher]: Unpredictable?

[Interviewee C]: Yes, but let me explain. When we say “I accept” or not, it's whether she experiences well the fact of being....

[Interviewee A]:...of being pregnant.

[Interviewee C]...being pregnant or not, because we have patients who, throughout the pregnancy, will not have initiated an abortion process, a termination of pregnancy, and who maintained the pregnancy, but ultimately experienced the pregnancy badly....

[Interviewee A]:...... and that they project themselves...

[Interviewee C]: So, there are some who don't project, some who can project a little bit. And then some have anxieties... So, it's more the psychologist who will talk, but we meet with patients who are in a process of anxiety and somatization of certain... certain symptoms that are indicative of difficulties.

We take that into consideration so that we can accompany the parents afterwards, once the mother has given birth... to be able to accompany and observe the establishment of the mother-child bond in the first days, even if we are not able to do it properly... but that's it... for us, it's also important to know that (Hospital - 2 FRA).

When it comes to the outcome of childbirth and women's reaction when they experience it, France presents quite demarcated logic instituted in childbirth, as well as a criticism of the practices of obstetric violence, as can be confirmed in the following statements:

And on Saturday morning, they had a meeting at the service to choose...anyway...to go through all the documents related to my pregnancy and, I guess, to make decisions. I think they decided at that point to do the C-section. Suddenly I see a gynecologist that I didn't know come into my room and say: “Well... listen... for us your baby is too small, so we are going to do a c-section. In half an hour we will start.” You don't even have time to think. So, imagine... I have never had an operation in my life. I was very afraid of having an operation, very afraid of a cesarean section (Mother/FRA/01).

She gave birth by cesarean section. For her, it was a tragedy exactly because she had never thought of having a cesarean delivery. I ask her if in the preparatory course they had not approached the theme of C-section, and she says that it is mentioned, but she would never have imagined that this would be the (report of an individual 2 interview/mother/FRA/0).

In Brazil, this understanding varied and was strongly associated with the logic reinforced by the professionals, women's financial conditions, and access to services:

[Researcher] You told me that your doctor told you in the third month that normal birth was not possible...

[Mother 2] Yes, so. And, well, when she told me, I'll be very honest, I didn't even suffer because then I could say: it was the doctor who told me. Laughter

(everyone laughs)

[Mother 2] So I didn't suffer. It was a very good pregnancy, but I was very afraid. So, when she said: “It's a cesarean section”, I said: “Okay! [Ah, gee, it makes no difference to me! Laughter

[Mother 4] I had no choice, right? Because it was a public service, I didn't have that choice. Unless the delivery was complicated enough to be a cesarean. In my case, in the public service, the delivery is normal (Social 2 - 2 BRA).

Besides the aspects exposed, the reality instituted for births, and the process of accompanying women differ in each country. In the analysis of this step, aspects involving the autonomy and protagonism of women were addressed, revealing fragility also in different ways, in the two countries. In Brazil, for example, this is confirmed in the existing institutional protocols in the services as revealed in the following statements:

[Professional 06] And there are also many that when they are hospitalized to leave... the child-friendly hospital has breastfeeding. So, in order for the mother to leave, she needs to breastfeed, she needs to give the breast. Yes, the mother doesn't want to. You see that the mother doesn't want to, it's no use. But the doctor comes and says: ”if you don't breastfeed, you won't leave.

[They speak at the same time] Doesn't she have the right to choose? not to want to give the breast? and my right? I want to give her a bottle!

[Professional 06] and ask: do you want to breastfeed? do you want to breastfeeding? it is her right! And then it seems that to go away she has no right.

[Professional 04] Even if the hospital is child-friendly, it is her right to choose or not to breastfeed.

[Professional 05] In this case, the psychology and social services are called in to see if there is a link. After all, she doesn't want to give the breast (Hospital - 01 BRA).

In the French context, the people interviewed reveal in their discourses that breastfeeding is a woman's choice. However, they recognize that there are strong incentives on the part of professionals in preparatory courses to breastfeed. Moreover, French women seem to be more confident about their desires regarding the care of their bodies. This aspect can be confirmed by the following statements:

[Professional W]: [...] when there is a discussion among the doctors in the morning, when we present the documentation, if, for example, a patient wants a C-section because there is minimal risk, but the protocol says “attempted vaginal delivery,” some doctors don't listen to the patient's words. The protocol says, “it will be vaginal,” it will be vaginal. Do you agree with me?

[Professional X]: Yes, and I think that, in fact, what is very important is to have had a real discussion between a professional and a patient... (Hospital - 01/FRA).

[Professional06]: Refers that professionals try to listen to women, but in the end, often, the justification for the actions taken is always based on the non-exposure to medical risk (individual interview report/PRO/FRA/03).

Despite the context evidenced above, other aspects related to the organization of services and professional practices are outlined, similarly to what occurs in Brazil, sustained by protocol actions, with intense medicalization of actions and interventionist practices.

## Discussion

In this study, the debate related to the feminine condition starts from the recognition that each woman's experience is unique; therefore, since the study is based on the reality of two countries, it was sought to constantly adopt an exercise of relativizing generalizations.

Women's sexual and reproductive health is on the agenda in Brazil and France, although with different approaches and developments. It is based primarily on the idea of individual freedom and gender equality. It seeks to combat the violation of rights from the visibility of different aspects, such as prevention and treatment of sexually transmitted infections, debate on sexual violence, termination of pregnancy, strategies to minimize maternal and neonatal mortality, and access to contraception and qualified obstetric care (Code de la Santé Publique, 2016). Feminist struggles, marked by strands of its waves, contribute to critical-reflexive deepening around women. In this process, the unfolding of these struggles demonstrates a political commitment that will influence the production of knowledge related to sexism, gender, social relations of sex, reproductive health, reproductive rights, and patriarchy, among others (Antunes, 2020).

The theoretical-methodological framework of the French institutional social clinic provides devices that enable the exercise of reflection on the constitutive processes of the institution of sexual and reproductive health as well as the identification of its contradictions. In the institution, the relationship between the instituted and instituting forces provokes a lot of the dynamics of its functioning, promoting constant changes, and the institutionalization (Lourau, 1970). However, it is apprehended that the institution is neither limited to its instituted, in this perspective of thought, nor to the sexual and reproductive health of women. It is transformed by the invention of new practices, new devices, and, often, in the renewal of organizational indicators in a struggle to stay alive.

The history of Western society is heavily influenced by patriarchal cultures. In this process, the Greeks, Romans, Jews, and Christians built a complex representation of women and motherhood. Patriarchy strongly influences the role of women in society, the maternal role, decisions, conceptions, and even the outcomes of childbirth. The nineteenth and twentieth centuries are remarkable in driving the glorification of motherhood, and the female body assumes the delineation of a social body that is used for reproduction (Knibiehler, 2012). In addition, capitalism promotes important demarcations in society and women's lives.

As highlighted in the results of the present production, the data produced in Brazil revealed that the logic of compulsory motherhood is strongly demarcated in its culture. It is understood that there are different reasons for the reinforcement of this reality, among which are the very formation history of the country as a colony of exploitation that reproduces patriarchal logic imposed by a colonizer, the social role of the mother, economic fluctuations, and dominant values. All these elements contribute to the delineation and reinforcement of the predominant feminine condition in the country. Through the years, patriarchy has had mechanisms to stay alive, and Brazil strongly reveals the consequences of its action in the society (Diniz et al., 2017; Antunes, 2020).

In France, there is a consistent analysis of parenting, feminist issues associated with sexual and reproductive health, motherhood, and sexual education in schools, as well as on interventionist practices developed by women's care professionals (Code de la Santé Publique, 2016). They often report on the existence of an asymmetry in the interaction between women and professionals. Professionals of assistance, in exercising their power, may use practices and speeches to censor, encourage, make invisible, misrepresent, value, or devalue the actions of service users (Silva et al., 2021).

It is possible to say that, in some way, there is a rapprochement between the two countries, in what involves the criticism regarding the monopoly of the discourse on women's bodies and health (Coulm and Blondel, 2013; Thomas, 2017; Betrán et al., 2018; Boerma et al., 2018; Grilo Diniz et al., 2018; Lansky et al., 2019). It is also possible to verify that in both countries there is an ambivalence in the attention to mothers and babies, which is revealed in the existence of a health organization that intends, on the one hand, to protect them through regulation of their behavior materialized in the establishment of an administrative surveillance (Molina, 2014; Silva et al., 2021).

The reality of Brazilian maternal and child health has been widely signaled in analyses developed in a global context, especially due to quantitative indicators related, for example, to unnecessary surgical deliveries, obstetric violence, and maternal and infant mortality (Betrán et al., 2018; Boerma et al., 2018; Gomes et al., 2018; Grilo Diniz et al., 2018; Lansky et al., 2019). The marked social and gender inequalities in the country, as well as the organization of the Brazilian health system, which is also critically analyzed, tend to influence the numerical data (Bottallo, 2021; World Health Organization, 2021).

In a global context, rates of medically unnecessary and potentially harmful surgical interventions in childbirth are increasing amid inequalities in access and are likely to continue rising until 2030 (World Health Organization, 2021). Discrepancies in the access show worrying data; after all, the intervention surgery could be essential and save lives; however, in less developed countries, 8% of births were by cesarean section, while in Latin America and the Caribbean, the rate of births reach 43% (World Health Organization, 2021). The causes of these developments vary among and within countries and may be due to political issues linked to health financing, cultural impositions, established practices, percentage of premature births, and quality of healthcare (World Health Organization, 2021).

Another point that emerges from the statements of the research participants and that gives visibility to the debate related to the female condition as a demarcator of sexual and reproductive health is abortion. In Brazil, despite being widely performed and the known relationship with the indicators of maternal death, it does not show advances in actions aimed at its guarantee. Due to its magnitude and persistence, abortion is a public health problem (Cardoso et al., 2020). The legislation of the country authorizes its accomplishment only in specific conditions, as in cases of pregnancy resultant from sexual abuses, when it puts in risk the life of the woman and when the fetus is anencephalic (Procedimento de Justificação e Autorização da Interrupção da Gravidez nos casos previstos em lei, 2020). Health professionals, when addressing the problem posed in the results of this research, confirm that abortion in the country is criminalized and point out their strategies to face the demand so frequent in the service. This criminalization not only kills but also persecutes and does not legitimize the right of women to choose when they will be mothers or how to raise their children, turning them, therefore, into criminals (Antunes, 2020). The Brazilian reality clearly reveals that the patriarchal narrative is still dominant and demonstrates its strength over the logic instituted in society regarding the condition of women. One cannot fail to register the violations and retrogressive actions of the current Brazilian governmental management, in what includes women's sexual and reproductive rights. This is revealed not only by the resistance to the world agendas related to women but also by the violation and negligence operated by the current governmental representations in what involves the fight for access, equality, and inclusion (Diniz et al., 2017; Antunes, 2020; Cardoso et al., 2020).

The data on abortion in Brazil are not accurate because of the availability of data only linked to the public sector, and mortality data depend on the investigation of death. Data numbers point out that between the years 2008 and 2015, an average of 200,000 hospitalizations/year had abortion-related procedures. Between 2006 and 2015, 770 maternal deaths with abortion as a basic cause were found in the Mortality Information System (Cardoso et al., 2020).

In France, voluntary termination of pregnancy is guaranteed by law (Code de la Santé Publique, 2016). The first objective of the legalization of abortion in this country in 1975 was to reduce the mortality and suffering of women who underwent clandestine^4^ abortions. The law (article L.2212-1 of the country's Public Health Code) allows any pregnant woman, adult or minor, who does not want to continue a pregnancy to have one legally performed and accompanied by trained professionals. In addition, the procedure can be performed on minors, with or without parental consent, as long as they are accompanied by an adult. Obviously, to perform the procedure, there are specific rules defined in the law, and it occurs under the accompaniment of a physician or sage-femme (Ministère des Solidarités et de la Santé, 2022). The debate on women's sexual and reproductive rights in France, which is not centered only on the interruption of pregnancy, makes issues related to health determinants more visible. Such a scenario enables the offer of an open dialogue about the processes demanded by women, as well as a more qualified assistance (Audibert, 2016).

Another element of the study that provides clues to the delineation of the female condition, and sexual and reproductive health is in the active force of the organizational models of services and their relationship with the professional practices developed. They reveal that women admitted to prenatal, childbirth, and puerperium care services suffer the consequences of the domination of their bodies. It is possible that this also occurs as a result of the pressures of financial logic on the functioning of the services and on professional practices. The experience of surgical deliveries experienced by the women participating in the research can exemplify this information. The statements reveal, for example, that in Brazil, C-section birth tends to be naturalized according to a woman's social/economic/educational level, while in France it is strongly associated with a woman's failure.

The discourse of Brazilian women, especially those from more privileged social backgrounds, reflects their process of subjection, as well as the process of domination of their bodies by professionals. In this country, even women belonging to the less privileged social environment tend to be subjected to the puerperal pregnancy process. They, different from the more privileged ones, face greater difficulties in accessing the healthcare network, in establishing contact with the professional that accompanies them, and with the service that receives them (Bottallo, 2021). Professionals, not rarely, place without any discomfort their perception that they are the holders of knowledge related to their bodies which, consequently, removes the need for women to participate in decisions related to their own bodies and the birth of their own children.

The results also reveal that French women, when experiencing deliveries with surgical outcomes, carry the feeling of having failed and are bothered by the passivity in the procedures employed in the conduction of childbirth, as well as by the manipulation of their bodies by professionals. All this is related to the reality of the feminine condition and the understanding of body autonomy. Moreover, the data produced in France reveal elements such as dissatisfaction with the type of care received in a vaginal birth, which promotes, in some cases, mobilization to deviate from the established logic of hospital births in search of greater autonomy in the childbirth process (Silva et al., 2021).

In the debate proposed by this article, another aspect revealed by the participants highlights how the organization of the service of the two countries tends to influence the professional's performance profile, the degree of interventions, the discourses to guide/convince women, the respect for the autonomy of these women, and the actions offered in the care. This reality is linked to innumerous factors, especially the management model and the financing of health actions that will reveal a rationalization of costs and the development of increasingly controlled practices (Lesieur et al., 2018; Milcent and Zbiri, 2018; Entringer et al., 2019).

Regarding breastfeeding, for example, Brazilian healthcare services tend to follow the protocols in force; in some cases, when a woman does not wish to breastfeed her baby, the service routine overrides the women's wishes, generating frustration. In France, the statements of the group of participants show that the agenda concerning this point seems to be better established, as it tends to seek to meet the wishes of a woman who participated in childbirth preparation sessions, among which specificities about breastfeeding are addressed. It is necessary to consider the fact that the practices and speeches of professionals tend to influence, although in different ways in each researched reality, the desires of women and families. The progression or regression of this is related to the degree of implementation of medical actions in healthcare (Silva et al., 2021). In this aspect, it is apprehended that a qualified and sensitive follow-up to the women's personal demands is salutary for the guarantee of their autonomy and protagonism. In the same way, preparation for childbirth has a great influence on the degree to which women come closer to the services and the established logic.

## Concluding Remarks

This study was interested in giving visibility to the agenda that involves the feminine condition and sexual and reproductive health of women. It revealed elements that mark the reality of Brazil and France from the reading of the female body as the one loaded with symbolic constructions and social symbols, which, to some degree, can be (un)visualized, (un)criminalized, and (non)violated in the exercise of their reproductive health. These subjective bodies need to resist the unfolding of patriarchal actions that tend to alienate social practices and, sometimes, relationships established between individuals.

Forces that act in the consolidation of policies and practices involved in guaranteeing women's sexual and reproductive rights show advances and, at the same time, contradictions made visible in the analysis of the relationships between the feminine condition and the health institution. The understanding of conceptions about life, death, satisfaction, suffering, and social policies is fundamental to guiding the study. This relationship involves right to privacy, self-determination, freedom, and individual autonomy, and needs to overcome the interference of the state in the search for overcoming discrimination, coercion, violence, and domination of the bodies.

The results reveal, in both countries, aspects about the social role of women's bodies, the established logic that is reflected in the decision to have children and in motherhood, the autonomy of women over their own bodies, the central values linked to the interruption of pregnancy, and the professional practices used in prenatal, delivery, and postpartum care. In general terms, the French reality questions the Brazilian reality, because Brazil is a country strongly marked by the naturalization of procedures that deprive the woman's autonomy. In this way, based on the elements addressed in this study, the French reality is questioned in what includes C-section birth. This is revealed in the practices adopted by professionals and services, especially in the preparation of a woman in the elements that contribute to the construction of what is expected by women in the outcome of childbirth and the influence of professional practices and discourses in this process.

The use of the theoretical-methodological framework of the institutional social-clinical offered paths for the use of tools and the construction of devices that instigate the institutions' speech and stimulate the reflexivity of the people involved. The exercise of questioning oneself about the implication of the subjects in the institutions, for example, values the affective, existential, and professional dimensions and allows us to walk toward the comprehension of the existing contradictions. The theoretical-methodological framework also offers support in the recognition of the mechanism of the actions of power in care practices, which can be camouflaged in well-intentioned, more efficient, and safer practices. Thus, it is apprehended that active forces, such as those coming from feminist movements and social movements connected with the agenda, which have historically given answers, will continue to act questioning the reality and claiming for changes.

## Data Availability Statement

The raw data supporting the conclusions of this article will be made available by the authors, without undue reservation.

## Ethics Statement

The studies involving human participants were reviewed and approved by University of São Paulo Clinical Hospital of the Ribeirão Medical School. The patients/participants provided their written informed consent to participate in this study.

## Author Contributions

All authors contributed to manuscript revision, read, and approved the submitted version.

## Funding

CY Foundation, Montfort Research Institute e University of São Paulo/PROEX - N° AUXPE: 0451/2021 - N° Processo: 23038.010138/2021-28.

## Conflict of Interest

The authors declare that the research was conducted in the absence of any commercial or financial relationships that could be construed as a potential conflict of interest.

## Publisher's Note

All claims expressed in this article are solely those of the authors and do not necessarily represent those of their affiliated organizations, or those of the publisher, the editors and the reviewers. Any product that may be evaluated in this article, or claim that may be made by its manufacturer, is not guaranteed or endorsed by the publisher.

## Footnotes

^1^According to Beauvoir, patriarchy is an arrangement of dogmas, unquestionable ideas, and laws that carry symbolic and physical violence, suffering, and guilt. People who are interested in maintaining their privileges of gender, sexuality, race, class, and age administer this arrangement.

^2^Title of study: Birth as a biopsychosocial process: a collaborative, interdisciplinary study of the effects of practices and discourses on perinatality on becoming parents in Canada, France, and Brazil.

^3^Not all the individual interviews conducted in France were transcribed. For those not transcribed, summarized texts were used.

^4^In her speech, Veil turns to the situation of women and argues how the legalization of abortion would favor motherhood, thus being a factor in the protection of all women who, in a given circumstance, need to abort, guaranteeing them health to be mothers one day (Assemblée Nationale Française, 2015).
